# Electrochemical Performance of Thick-Film Li(Ni_0.6_Mn_0.2_Co_0.2_)O_2_ Cathode with Hierarchic Structures and Laser Ablation

**DOI:** 10.3390/nano11112962

**Published:** 2021-11-04

**Authors:** Zelai Song, Penghui Zhu, Wilhelm Pfleging, Jiyu Sun

**Affiliations:** 1 Institute for Applied Materials—Applied Materials Physics (IAM-AWP), Karlsruhe Institute of Technology, Hermann-von-Helmholtz-Platz 1, 76344 Eggenstein-Leopoldshafen, Germany; songzelai@foxmail.com (Z.S.); wilhelm.pfleging@kit.edu (W.P.); 2 Key Laboratory of Bionic Engineering (Ministry of Education, China), Jilin University, Changchun 130022, China; sjy@jlu.edu.cn

**Keywords:** lithium-ion battery, cathodes, NMC 622, multilayer, hierarchical structure, laser structure, cyclic voltammetry, impedance spectroscopy

## Abstract

The electrochemical performance of lithium-ion batteries is directly influenced by type of active material as well as its morphology. In order to evaluate the impact of particle morphology in thick-film electrodes, Li(Ni_0.6_Mn_0.2_Co_0.2_)O_2_ (NMC 622) cathodes with bilayer structure consisting of two different particle sizes were manufactured and electrochemically characterized in coin cells design. The hierarchical thick-film electrodes were generated by multiple casting using NMC 622 (TA) with small particle size of 6.7 µm and NMC 622 (BA) with large particle size of 12.8 µm. Besides, reference electrodes with one type of active material as well as with two type of materials established during mixing process (BT) were manufactured. The total film thickness of all hierarchical composite electrodes were kept constant at 150 µm, while the thicknesses of TA and BA were set at 1:2, 1:1, and 2:1. Meanwhile, three kinds of thin-film cathodes with 70 µm were applied to represent the state-of-the-art approach. Subsequently, ultrafast laser ablation was applied to generate groove structures inside the electrodes. The results demonstrate that cells with thin-film or thick-film cathode only containing TA, cells with bilayer electrode containing TBA 1:2, and cells with laser-structured electrodes show higher capacity at C/2 to 5C, respectively.

## 1. Introduction

Lithium-ion batteries (LIBs) are widely used in daily life for electric vehicles, mobile applications, and electric energy storage devices [1,2]. Although they had been making our lives convenient and clean, their charge and discharge capacity needed to be improved with the development of electronic devices [3]. The cathode and anode can influence the electrochemical performance of LIBs. Lithium-ion migration in electrodes is the necessary electrochemical process to store energy [4]. LiNi_1−x−y_Mn_x_Co_y_O_2_ (NMC) materials are very promising candidates for energy storage due to their high energy density and excellent charge-discharge capacity compared to LiCoO_2_, LiMn_2_O_4_ (LMO), and LiNiO_2_ [5], and NMC 622 is a cathode material with a higher specific discharge capacity of 170 mAh/g (2.5–4.3 V) than NMC 111, LFP, and LCO [6]. Increasing the electrode thickness can improve LIBs’ areal capacity, but the performance at higher C-rates is limited as a result of the degradation of the thick-film electrode [7]. The poor lithium-ion diffusion kinetics in cells with thick-film NMC electrodes lead to poor rate capability, capacity loss at high current rate, and low capacity retention during long-term cycling due to an increased cell polarization [8]. Increasing the thickness or reducing porosity of thick-film electrodes can achieve high specific energy [9]. Therefore, other methods such as laser processing is of great importance for batteries with thick-film electrodes to achieve high energy density as well as high power at the same time.

The application of ultrafast laser processing in manufacturing of LIBs has attracted more and more attention. Laser electrode cutting can improve the qualities of the cut edges, forming less debris while shortening the processing time [1], and therefore is advantageous for industrial mass production. Recent studies show that electrochemical performances of LIBs containing laser-structured electrodes are enhanced using different laser patterning [10], such as lines [11], grids [12], and holes [13]. Cells with laser-structured NMC and LMO electrodes exhibit increased capacity retention and longer cycle life-time while being cycled at 1C [14]. In comparison to cells with unstructured electrodes, the lithium-ion diffusion kinetics in cells with structured electrodes are higher due to new diffusion pathways along the sidewalls of channel or grid structures. Besides, the cell polarization of cells with structured electrodes is decreased. These advantages lead to simultaneous enhancement of power and energy densities of electrodes [9,15]. Therefore, their charge/discharge rate and specific energy density at cell level can be greatly increased [11,16]. For example, a recent study showed that cells containing thick-film laser-structured NMC 622 electrodes exhibit higher discharge capacities at C/2 to 5C compared with the ones without structures [17].

The cycle stability and the energy density of LIBs can be influenced by the thickness of electrodes [18]. The power of LIBs is reduced dramatically because the total internal resistance is strongly influenced by both ionic resistance in pores and charge-transfer resistance for lithium intercalation [19]. The additives of polyvinylidene difluoride (PVDF) binder and conductive carbon black in electrode can influence LIBs’ electrochemical properties [20,21]. The high-energy LIBs can be achieved by the ultra-thick cathodes with thickness of up to 700 µm [22]. Besides, suitable electrolyte compositions, for example, with EC:EMC (3:7) as solvents and suitable additives, are needed to improve the cycling performance and reduce the volume expansion [23]. The optimal porosity of thick-film electrodes after calendering should be adjusted to 30% to 40%, in order to increase electric contact between active material particles as well as between active materials and carbon black [24]. Another way to improve the electrochemical performance is by using blended active materials. Suitable materials blending leads not only to balancing the constituents’ properties but also can surpass the performance of each constituent [25]. Nanomaterials are much more reactive than submicron particles such as LiMn_1/3_Ni_1/3_Co_1/3_O_2_ [26]. Two cathode materials, LiNi_x_Co_1-x-y_Al_y_O_2_ and LiMn_2_O_4_, blended by physical mixtures minimize the shortcomings of the parent materials, and can be tailored to get a high power density or higher energy LIBs with the high stability and low cost [27]. The blended cathode displays an average electrochemical performance of the two cathode materials according to the simulation [28]. The material with high rate capability can help the other material to retain 75% capacity relative to the initial value during the long-term test [4]. Intermediate crystallite size NMC acquired by ball milling shows better performance, such as high rate capabilities compared to the base material [29,30]. The simulations reveal that coin cells with bilayer cathodes exhibit promising performance with 39% higher discharge capacity at 2C and an improved lithium-ion diffusion due to the small particle size [31].

In this paper, the electrochemical performance of hierarchical cathodes containing NMC 622 powders with different particle sizes is investigated. The multilayer thick-film electrodes with 150 µm thickness were manufactured by multiple casting using slurries containing NMC 622 powder with different particle size, while the thicknesses of electrode containing polycrystalline NMC 622 (BA) with large particle size of 12.8 µm and NMC 622 (TA) with small particle size of 6.7 µm were set at 1:2, 1:1, and 2:1. After drying and calendering, different electrodes were structured using femtosecond (fs) fiber laser to generate line structures. Rate capability test, long-term test, cyclic voltammetry, as well as electrochemical impedance spectroscopy (EIS) were used to characterize coin cells assembled with different electrodes.

## 2. Materials and Methods

### 2.1. Cathode Materials

In order to manufacture cathodes with hierarchical structures, two commercially available NMC 622 powders were used in this work, which are single crystalline NMC 622 (TA, supplier 1) with small particle size of 6.7 µm, and polycrystalline NMC 622 (BA, supplier 2) with large particle size of 12.8 µm. The particle sizes of TA and BA were characterized using laser scattering particle size distribution analyzer (LA-950, Horiba Ltd., Kyoto, Japan). Polyvinylidene fluoride (PVDF) Solef^®^ 5130 (Solvay Specialty Polymers, Paris, France) was used as binder, while carbon black C-NERGY Super C65 (Imerys G & C Belgium, Willebroek, Belgium) and KS6L Graphite (Imerys G&C Switzerland Ltd., Bodio, Switzerland) were applied as conductive agent and compaction aid, respectively.

PVDF powder was dissolved in N-methyl-2-pyrrolidone solvent (NMP, Merck KGaA, Darmstadt, Germany) with a weight proportion of 1:10 and was homogenized using a planetary mixer (SpeedMixer DAC 150 SP, Hauschild GmbH & Co. KG, Hamm, Germany) with rotation speeds of 1000 to 3000 rpm for 30 min. Afterwards, electrode slurry was prepared with 92 wt.% NMC 622, 3 wt.% carbon black, 2 wt.% KS6L graphite, and 3 wt.% PVDF (dissolved in NMP) using SpeedMixer with rotation speeds of 1000 rpm to 3500 rpm for 1.5 h. During the mixing process, extra 3 g NMP was added into the cathode slurry to adjust the solid content of slurries to 66.7 wt.%.

Afterwards, cathode slurry was casted on a 20 µm aluminum foil on the film coater (MSK-AFA-L800-LD, MTI Corporation, Richmond, CA, USA) with a coating speed of 5 mm/s. A doctor blade (ZUA 2000.100, Proceq, Schwerzenbach, Switzerland) was used to control the wet film thickness of the cathode by adjusting the gap between the blade and current collector. To make the cathodes with hierarchical structure, the basis film was firstly casted and dried for 3 h at 90 °C. Then, the heater was turned off and the film as well as coater were cooled down to room temperature. The second film was subsequently casted on top of the previous film.

Three different types of cathodes were manufactured, which are with single type of NMC 622 powder; mixture of two NMC 622 powders in one film; and hierarchical structures with two layers containing different NMC 622 particle sizes. The total film thickness was kept constant at 150 µm, while the ratios of hierarchical films were set to 1:1 and 1:2 (2:1). In order to distinguish the position of different layers in the hierarchical films, the following abbreviations were used: TA represents the film with small NMC particles, while BA means the film with large NMC particles. According to the position of bottom film and upper film, different notations are used. For example, TBA indicates that TA layer with small particle size is at the bottom (near current collector), while BA layer is on the top. Therefore, BTA suggests that in this electrode, BA is the bottom layer and TA is the top layer. BT means that two NMC 622 powders were added simultaneously into the slurry during mixing process, thus no multilayer exists in final electrode. Besides, thin-film electrodes with 70 µm in thickness with single type of powder were manufactured as references representing the state-of-the-art approach.

The calendering procedure can achieve an overall homogenous cathode film thickness and improve the particle contact inside the cathode. According to other research, the optimal porosity of LIBs should be in the range of 30–40% [24]. Thus, the porosity of different cathodes in the work were adjusted to about 35% after calendering. The cathode film was calendered by an electric precision rolling press (MSK-2150, MTI Corporation, Richmond, CA, USA) at room temperature with a forward rolling speed of 35 mm/s. The porosity of electrodes is defined as follows [9]:

(1) $$Porosity=\frac{V-W\left\{\;\frac{C_{NMC}}{\rho_{NMC}}+\frac{C_{Super\;C}}{\rho_{Super\;C}}+\frac{C_{\mathrm{KS}6L\;\mathrm{Graphite}}}{\rho_{\mathrm{KS}6L\;\mathrm{Graphite}}}+\frac{C_{PVDF}\;}{\rho_{PVDF}}\right\}}{V}\times 100\%$$

where *V* is the volume of the composite electrode without current collector, *C* is the mass ratio of each material in the composite electrode, *W* is the weight per area, and *ρ* is the density of each material.

### 2.2. Laser Structuring

An ultrafast femtosecond fiber laser (Tangerine, Amplitude Systèmes, Pessac, France) with a pulse duration of 380 fs and an operational wavelength of 515 nm (M^2^ < 1.2) was applied to generate laser structures and cut cathodes into 12 mm circles for coin cell assembly. The laser processing was performed in ambient air. Line structures with 200 µm pitch and with depths down to the current collector were generated, with a laser repetition rate of 500 kHz and an average laser power of 2.5 W. The laser scanning speed was kept constant at 500 mm/s, while the number of scan passes varied from 8 to 23 with regard to the active materials, film thickness, as well as hierarchical structures.

Cross-sectional analyses of electrodes using different scan passes were performed in order to find appropriate structuring parameters. Samples with a size of 2 × 1.3 cm (width × height) were cut from the cathode film using the same laser. The laser-structured samples were held vertically with two glass sheets and fixed at the bottom using a plastic clip. They were placed in a holder filled with embedding solution consisted of resin, hardener and fluorescent powder. After 16 h drying, the samples were ground by a grinder-polisher (Ecoment 3, Buehler Ltd., Esslingen, Germany) and polished using a metallographic laboratory polisher (Saphir 350, ATM Qness GmbH, Mammelzen, Germany).

A scanning electron microscope (SEM, Phenom XL G2 Desktop, Thermo Fisher Scientific Inc.-FEI Deutschland GmbH, Dreieich, Germany) was used to obtain the images of two NMC 622 powders, the electrodes with hierarchical structures, and laser-structured electrodes. The crystal structure and the surface chemistry of electrode materials were investigated by X-ray diffraction (XRD) measurement. The XRD measurements (Empyrean, Malvern Panalytical Ltd., Malvern, UK) were performed using with CuKα radiation in 2θ range of 0–90° (200 s/step) at 40 mA and 40 kV.

### 2.3. Coin Cell Assembly

The laser cut electrodes (diameter 12 mm) were stored in a vacuum oven (VT 6025, Heraeus GmbH, Hanau, Germany) at 130 °C for 16 h. The cathodes were then assembled versus lithium foil in coin cells CR2032 in an argon-filled glove box with H_2_O < 0.1 ppm and O_2_ < 0.1 ppm (LABmaster sp, M. Braun Inertgas-Systeme GmbH, München, Germany). Each electrode was soaked in electrolyte for 30 min to be homogenously and completely wetted. The electrolyte consists of ethylene carbonate and ethyl methyl carbonate (EC/EMC 3:7), with 1.3 M lithium hexafluorophosphate (LiPF_6_) as conducting salt and 5 wt.% fluoroethylene carbonate (FEC) as additive, respectively. In total, 120 µL electrolyte were added into the coin cell. Polypropylene separator (Celgard, Charlotte, NC, USA) with a thickness of 25 µm and a diameter of 15 mm was used. After stacking, the coin cell was pressed together using an electric crimper machine (MSK-160E, MTI Corporation, Richmond, CA, USA).

### 2.4. Electrochemical Analysis

#### 2.4.1. Rate Capability Tests

A battery cycler (BT 2000, Arbin Instruments, College Station, TX, USA) was used to test the rate capability of coin cells. During charging, the so-called “constant current-constant voltage” (CCCV) method was applied, while only constant current (CC) was used for discharging. The C-rate was calculated based on the discharge time and applied current from the formation step at C/20. Then 1C and a specific capacity of 172 mAh/g were applied for the current calculation under different C-rates. At the beginning, 3 cycles at C/20 were carried out as the formation step, which ensures the homogeneous growth of solid electrolyte interface on the electrode. Then, 5 cycles were performed at C/10 and C/5. After this, the C-rate increased from C/2 to 1C, 2C, 3C, and 5C, and the coin cells were cycled 10 times at each C-rates. Finally, 5 cycles at C/5 was applied to test the capacity retention after fast charging/discharging. In order to analyze the irreversible capacity loss during long-term tests, coin cells containing thin-films were cycled at 1C for 100 cycles, while C/2 was applied for cells with thick-film electrodes for 40 cycles. The cut-off voltages were set from 3.0 to 4.3 V.

#### 2.4.2. Cyclic Voltammetry

After rate capability test, cyclic voltammetry (CV) test was performed using a battery cycler (BCS-810, BioLogic, Seyssinet-Pariset, France) in order to determine the redox reaction of coin cells during charging and discharging processes. A scan rate of 0.02 mV/s was applied, while the voltage range for CV was set from 3.0 to 4.3 V. For each coin cell, CV was repeated three times.

#### 2.4.3. Electrochemical Impedance Spectroscopy

Electrochemical impedance spectroscopy (EIS) was used to determine the impedance of coin cells with different kinds of electrodes. After formation, the coin cell voltage gradually increased from 3.0 V to a stable voltage at around 3.6 V, where EIS analyses were performed using a battery cycler (VMP3, BioLogic, Seyssinet-Pariset, France) in an oven with a constant temperature of 25 °C. The scanning range was from 1 to 0.01 Hz, while a sinus amplitude of 10 mV was applied.

## 3. Results

### 3.1. Characterization of Electrodes

**Figure 1:**
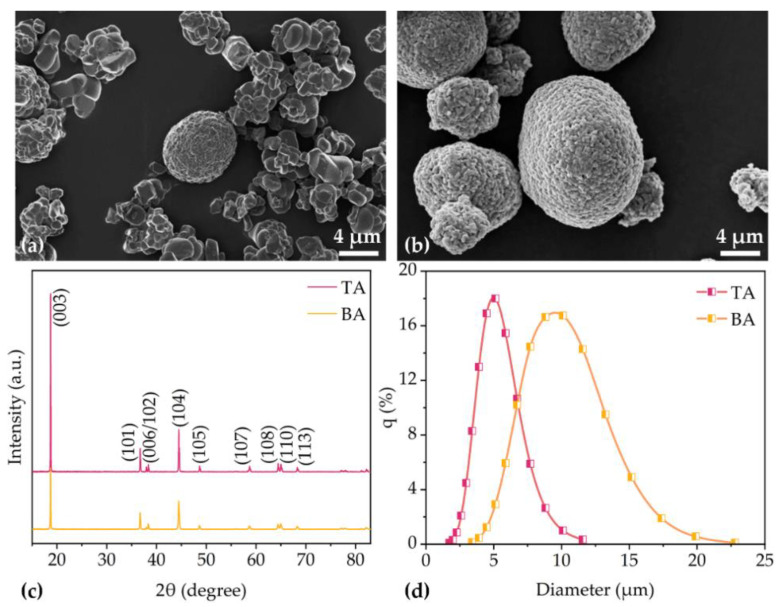
The SEM images of (**a**) TA NMC 622 particles and (**b**) BA NMC 622 particles. The (**c**) XRD patterns and (**d**) particle size distribution of two NMC 622 samples.

The SEM images of NMC 622 powders with small particles NMC 622 (TA) and big particle size (BA) are shown in Figure 1a,b. TA particles have diameters ranging from 1–6 µm, while BA particles have about 10 to 14 µm diameter and have spherical morphology. Figure S3 provides SEM images of the two NMC 622 particles with 20000 times magnification. The secondary particles and primary particles can be clearly distinguished. Laser scattering was applied to measure the particle size distribution of secondary particles, as shown in Figure 1d. TA NMC 622 particles have a D90 of 6.7 µm, which is about 1/2 compared to BA with D90 of 12.8 µm. Figure 1c presents the XRD patterns for two NMC 622 particles, demonstrating their purity as the single-phase layered materials. TA sample showed similar reflexes and reflex positions as BA.

The porosities of different cathodes decreased to 35% after calendering. The thicknesses of thin-film and thick-film electrodes are about 70 and 150 µm. The thickness ratios of TA and BA film inside hierarchical electrodes were set to 1:2, 1:1, and 2:1. The details of various cathodes are summarized in Table 1. Active mass loading is the mass of NMC 622 per unit area and areal capacity is calculated based on the mass loading.

**Figure 2:**
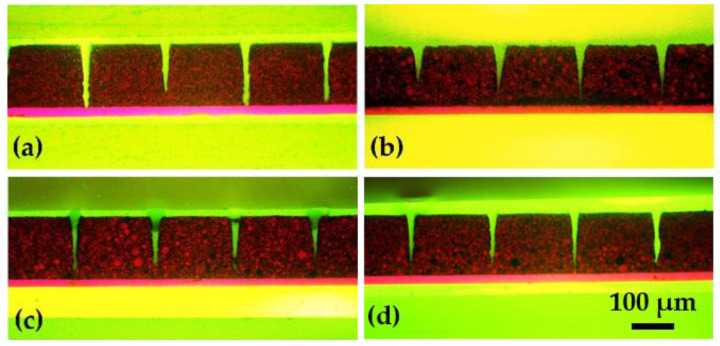
Microscopic images of thick-film electrodes in cross-sectional views. The number of laser scans has been adjusted to enable ablation down to the current collector: (**a**) TA, 14 scans; (**b**) BA, 22 scans; (**c**) BT, 15 scans; (**d**) BTA 1:1, 15 scans.

Figure 2 shows the cross-sections of laser-structured samples. For electrodes with different multilayers, the laser scan was adjusted to achieve channel structures reaching from electrode surface down to the current collector.

**Figure 3:**
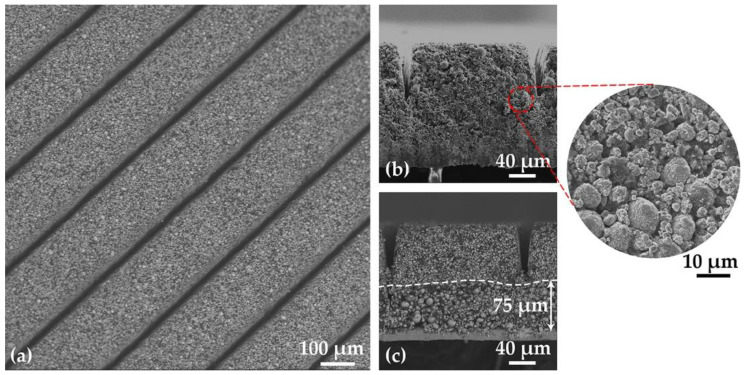
SEM images of (**a**) thick-film electrodes with BT from top view, and the cross section of thick-film electrode with (**b**) BT and (**c**) bilayer BTA 1:1.

The SEM images of laser-structured hierarchical cathodes are shown in Figure 3. In the first image, it is found that the channels are homogenous with a width of about 25 µm near the surface, which is consistent with the result from cross-sections. Figure 3b shows a BT film, in which NMC 622 powders with two different sizes are mixed together. No significant aggregation of the NMC 622 particles is observed and the two powders are mixed homogenously. Figure 3c shows thick-film hierarchical cathodes with BA NMC 622 at the bottom and TA NMC 622 on the top, a clear boundary is observed at about 75 µm.

The mass loss due to laser ablation includes the removal of active material, conductive additive, and binder. The active mass loading and areal capacity of laser-structured electrodes are summarized in Table 2.

### 3.2. Electrochemical Analysis

All NMC 622 electrodes were assembled vs. lithium in coin cells. The results from the rate capability test, cyclic voltammetry, as well as electrochemical impedance spectroscopy are presented in this chapter.

#### 3.2.1. Rate Capability Tests

**Figure 4:**
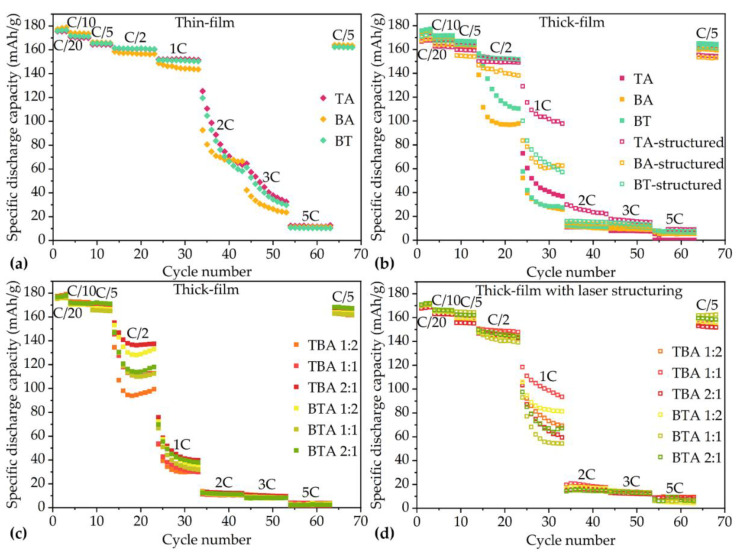
Rate capability tests of cells containing (**a**) thin-film electrodes with TA, BA, and BT. (**b**) Cells containing thick-film electrodes with TA, BA, and BT. Coin cells containing (**c**) unstructured thick-film multilayer electrodes and (**d**) structured multilayer thick-film electrodes.

The specific discharge capacity of the coin cells with various electrodes are shown in Figure 4. Coin cells with thin-film electrodes show higher discharge capacity from C/2 to 3C in comparison to cells with thick-film electrodes. However, at the discharge rates 2C and 3C, the discharge capacity of coin cells with thin-film electrodes decrease from 120 mAh/g to 30 mAh/g. At 5C, coin cells with thin-film electrode maintain about 10 mAh/g capacity.

During the formation step at C/20, the specific discharge capacities of all coin cells increase slightly. For example, for coin cell with BT electrode, its specific discharge capacity increases from 176 to 177 mAh/g, while coin cell with TA electrode achieves 173 mAh/g after the third C/20 cycle. For the bilayer electrodes, the electrode containing TBA 1:2 achieves 179 mAh/g capacity, while electrode containing BTA 1:2 reaches 176 mAh/g capacity after the last C/20 cycle. At C/10, the cell with BT electrode retains the highest specific discharge capacity of 172 mAh/g, while the cell with BTA 1:1 electrode shows the lowest specific discharge capacity of 165 mAh/g. When the C-rate rises to C/5, coin cell with BTA 2:1 electrode holds the highest capacity of 171 mAh/g, while the lowest specific capacity of 162 mAh/g belongs to the coin cells containing BA electrode. At C/2, coin cell with TA electrode has the highest specific discharge capacity of 152 mAh/g, while the lowest specific capacity of 98 mAh/g is the coin cell containing BA electrode. From 1C to 2C, all coin cells with thick-film electrodes exhibit a capacity decrease of 80% relative to the initial discharge capacity. The coin cell with TBA 2:1 electrode maintains the highest capacity of 38 mAh/h at 1C and 11 mAh/g at 2C, respectively. The coin cell with BT electrode maintains the highest capacity of 11 mAh/g, while coin cell with TA electrode shows the lowest discharge capacity of 8 mAh/g at 3C. For the discharge rates 5C, all coin cells with unstructured thick-film electrodes show almost no capacity.

Rate capability test shows that the specific discharge capacities of coin cells with unstructured thick-film electrodes drop from 160 to 10 mAh/g with increasing current rate from C/2 to 2C. On the contrary, coin cells with laser-structured electrodes maintain about 150 mAh/g, while the capacities of cells with unstructured electrodes continuously decrease from 150 to 110 mAh/g at C/2. At 1C, the discharge capacities of cells with laser-structured electrodes are at least 69% higher than that of cells with unstructured electrodes. This difference between the laser-structured and unstructured electrodes in coin cells increases with increasing current rate. For example, the discharge capacity of the coin cell with laser-structured electrode at 1C was five times higher than that of the unstructured one. Meanwhile, Figure 4d shows the discharge capacity of coin cells containing laser-structured hierarchical electrodes. In comparison to cell with unstructured electrodes, the cell with laser-structured electrode shows about 2–4% lower capacity at C/10. However, at C/2, the discharge capacities of cells containing laser-structured electrodes are about 4–46% higher than ones with unstructured electrodes. With increasing C-rates to 5C, the cells with laser-structured electrodes show at least two times higher capacity than those of the cells with unstructured electrodes.

#### 3.2.2. Cyclic Voltammetry

**Figure 5:**
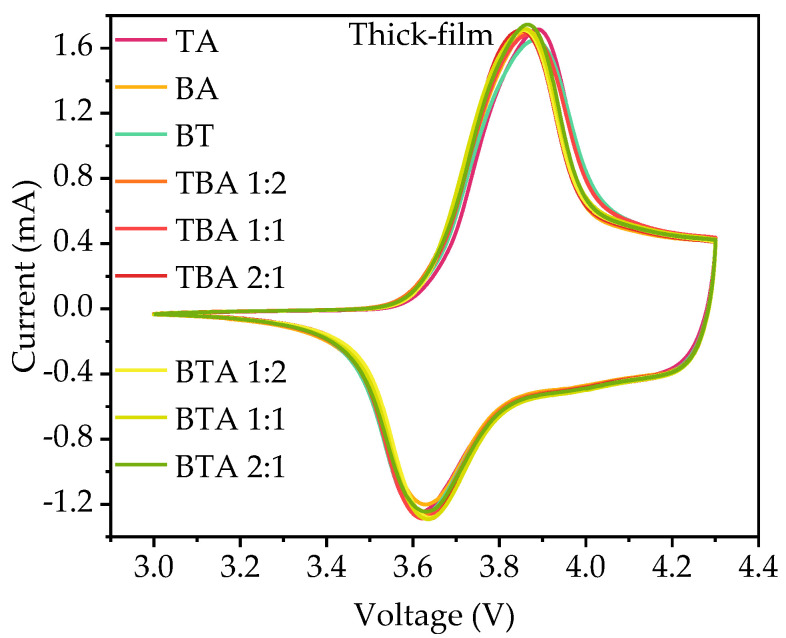
The CV measurements of the coin cells with unstructured single-layer and bilayer electrodes.

CV measurements were performed on coin cells with single-layer and bilayer cathodes to investigate the effect of hierarchical structures on the electrochemical reaction of the active material. Each coin cell was measured three times and the results overlap with each other as shown in Figure 5, which proves good reproducibility of the prepared bilayer electrodes. The recorded current signals formed a closed loop during charging and discharging. For bilayer cathodes, only one current peak appears during charging and discharging process, indicating the presence of an oxidation (Ni^2+^ → Ni^3+^ → Ni^4+^) during charging and reduction (Ni^4+^ → Ni^3+^ → Ni^2+^) during discharging. The oxidation peak shifts from 3.85 to 3.89 V, while the reduction peak shifts from 3.64 to 3.62 V.

#### 3.2.3. Electrochemical Impedance Spectroscopy

**Figure 6:**
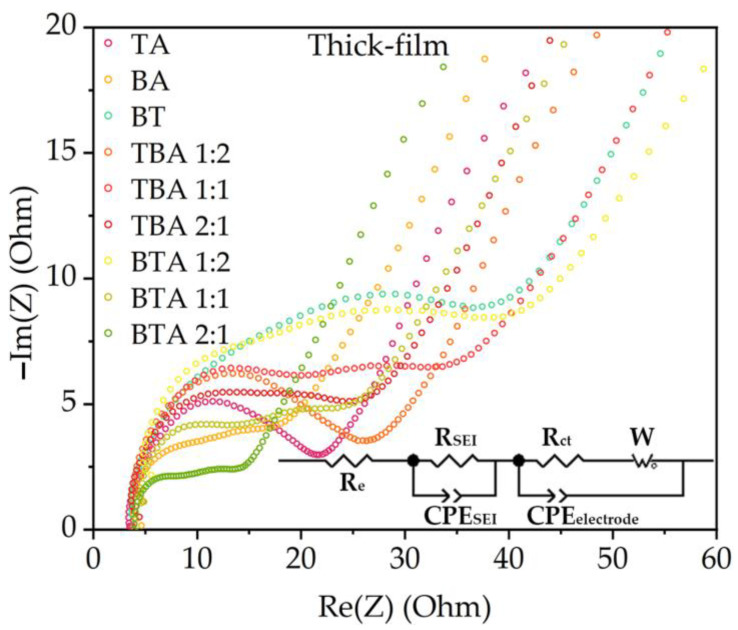
The EIS measurements of the coin cells with various hierarchical structured electrodes and the equivalent circuit model.

EIS is an effective technique to study the electrochemical reaction of the bilayer NMC 622 cathodes at different time scales [32]. Figure 6 shows the Nyquist plot of cells with unstructured single-layer as well as single-layer electrode. The complex Nyquist plot generally reveals a high-frequency semicircle and a low-frequency tail. The high-frequency semicircle is attributed to the interfacial charge-transfer processes, and the low-frequency feature is attributed to diffusion processes of lithium-ions in either the liquid electrolyte and in the bulk phase of the active material [26]. The EIS data were fitted by ZView using an equivalent circuit model as shown in Figure 6. The circuit element R_e_ represents series resistance of liquid electrolyte, separator, and bilayer electrodes. R_SEI_ and CPE_SEI_ are the resistance and capacity of the solid electrolyte interface (SEI), while R_ct_ represents the charge transfer resistance. A Warburg element (W) in parallel with constant phase elements (CPE) represents the diffusional process of lithium-ions inside the composite electrodes.

## 4. Discussion

### 4.1. Electrode Manufacturing and Laser Structuring

XRD measurement shows that TA sample and BA powders have similar reflexes and reflex positions, which indicates that the chemical composition as well as phase of BA and TA are equivalent. All the identified reflexes are in agreement with data of NMC from another study [29].

Multiple casting with different doctor blade height was used to manufacture bilayer electrodes. The separation of bilayer is clearly visible, as shown in SEM images and cross sections (Figure 2d and Figure 3c). However, it is observed that at the interface between two layers, the NMC particles in the top layer can infiltrate into the underlying layer. The binder is used to improve the mechanical strength and the adhesion of the cathode on the aluminum foil [21]. During the second casting, slurry with NMP was cast onto the first layer. Before the electrode is fully dried, part of the PVDF binder near the surface of the bottom layer could dissolve again into the NMP, causing mechanical instability near the interface of both layers and resulting in a relocation of NMC particles. When large NMC 622 particles are at the top, they will infiltrate deeper into the bottom layer due to gravity, as shown in Figure S1d–f. The BT electrodes with a mixture of both types of NMC 622 powders show a homogeneous particle distribution (Figure 3b) without agglomeration of the smaller NMC 622 particles.

The porosities of different cathodes were adjusted to about 35% in this work, since a porosity below 30% is very detrimental for the electrochemical performance of electrode, and porosities above 40% may generate electronic limitations [24]. With increasing electrode thickness from 70 to 150 µm, the active mass loading and areal capacity increases from 18 to 38 mg/cm^2^ and 3.1 to 6.6 mAh/cm^2^, respectively.

Figure S2 shows the surface of a BA-electrode close to a laser-ablated channel. The sidewall of the generated channel is smooth, indicating a sublimation of electrode materials from solid to vapor state without any evidence of melting. Different numbers of laser scans were applied during laser structuring of electrodes in order to achieve ablation through the entire composite electrode. BA electrode with large particle size and bilayer electrode with BA on the top need more scans in comparison to TA electrode with small particles, which means that the ablation depth per pulse for BA electrode is smaller than for the TA electrode. Since the laser fluence is constant by applying an average laser power of 2.5 W and a repetition rate of 500 kHz, the difference in ablation rate indicates that the ablation threshold fluence of BA NMC 622 with large particles is higher compared to TA NMC 622 with small particles. By mixing small particles into the electrodes, the laser processing time is decreased due to an increased ablation rate. Besides, Figure 2 shows that the channels of structured TA electrode are wider and rectangular, while BA electrodes have more Gaussian-like shaped channels with narrow width close to the current collector. For bilayer electrodes, it is found that the mass loss of laser-structured electrode is about 5% higher with TA layer on the top in comparison to bilayer electrodes with BA layer on the top. Thus, it is effective to reduce mass loss by using multilayer with large particle size in the top layer.

### 4.2. Electrochemical Analysis

#### 4.2.1. Rate Capability Tests

The electrochemical performance of LIBs depends strongly on the electrode composition and processing parameters [24], thus the mixing procedure as well as other components such as conductive agent and binder remain the same in this work. Lithium-ion migration in electrodes is the necessary electrochemical process to store energy for the battery [4]. The charge storage processes involve highly coupled, multiple transport phenomena that effect all electrode performance containing interfacial charge transfer, solid-state ion diffusion, electronic conductivity between and within particles, and diffusion of lithium-ions within the electrode [29].

Figure 4 shows the cells with BT have moderate discharge capacity between cells with TA and BA. The reason is that the blended electrode has the average electrochemical performance of two different positive active materials [28]. This phenomenon is also observed for cells with bilayer electrodes. With increasing thickness of TA as bottom layer, the discharge capacities of cells increase at 2C. The hierarchical structures and the position of TA and BA layer in cathodes play an important role in the electrochemical performance and will be discussed further.

With reducing the thickness of electrodes, the lithium-ion diffusion distance within electrodes is decreased [29]. Cells with thin-film electrodes show high discharge capacity during C/2, 1C, 2C, 3C, and 5C. Increasing the electrode thickness can indeed increase capacity at cell level, for example, cells containing unstructured thick-film electrodes have about 6.5 mAh/cm^2^ areal capacity, while cells with thin-film electrodes provide 3.1 mAh/cm^2^. However, the increasing of electrode thickness can enhance its energy density, but is accompanied by the significant loss of power density that results in the deterioration of rate capability [8]. For example, the discharge capacity of thick-film electrode containing BA decreases from 162 to 10 mAh/g at C/2 to 2C. The degradation of rate capability with increasing electrode thickness results from lithium-ion diffusion within the electrode [8]. The deterioration of energy density with cathode thickness and discharge rate increasing is due to the increase of the internal resistance and the damage of the mechanical integrity of the thick-film cathode materials [11]. Besides, insufficient wetting of electrodes can also result in rapid decrease of the discharge capacity at high current rates [9], therefore, during cell assembly, pre-wetting of electrodes and abundant amount of electrolyte was applied to avoid this issue.

The simulation from Chowdhury et al. [31] suggests that the cells containing bilayer electrodes with small particles next to current collector should have higher capacity at C-rates of 3C and 4C in comparison to cells with single-layer electrodes and bilayer electrodes with big particles close to the current collector. However, experimental studies in this work show that at C/2 to 5C, the position of layer with small or large particles has no influence on the electrochemical performance of the cells. Yet a difference in electrochemical performance is observed after laser structuring: cells containing structured electrodes with small particles next to current collector show higher capacity at C/2 and 1C in comparison to ones with big particles next to current collector. The cells containing NMC with an intermediate crystallite size of 86 nm own higher capacities as suggested by Malcolm et al. [29]. Figure 4b shows that a cell with TA has higher capacity compared to cells with BA and BT from C/10 to 1C. The beneficial effect of the smaller crystalline NMC 622 might due to the reduced diffusion distance of lithium-ions.

Cells with laser-structured thick-film cathodes have higher rate capability in comparison to cells with unstructured electrodes regardless of the C-rates [9]. This phenomenon is also observed in this work for all investigated cells. Geometric changes of thick-film electrodes by laser processing contributes to the decrease of electronic and ionic resistances, decrease of the tortuosity, and enhancement of lithium-ion diffusion without thermal damage, chemically negative reaction, or failure of the electrode structure [22]. The lithium-ion diffusion kinetics is increased due to channels inside the laser-structured electrodes filled with liquid electrolyte [10]. Channels in electrode can reduce the diffusion distance of lithium-ions in electrode pores filled with electrolyte by connecting pores with the free electrolyte in the cell.

**Figure 7:**
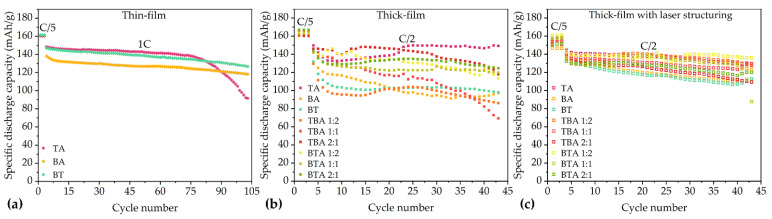
The results of long-term tests of the coin cells with different electrodes containing: (**a**) thin-film cathodes at 1C with 100 cycles; (**b**) thick-film cathodes at C/2 with 40 cycles; and (**c**) laser-structured thick-film cathodes at C/2 with 40 cycles.

From rate capability tests, it is concluded that cells with thin-film electrodes have a higher discharge capacity than cells with thick electrodes. Thus, the cells with thin-film electrodes were tested at 1C for 100 cycles, while cells containing thick-film electrodes were cycled at C/2 for 40 cycles in order to determine the long-term capacity retention. The results of long-term tests of the cells with different electrodes are shown in Figure 7. With regard to cells with thin-film electrodes, BT and BA own the highest capacity retention, with 86% after 100 cycles, while TA shows 62% capacity after 100 cycles. The discharge capacities of cells with unstructured thick-film electrodes deteriorate during long-term tests. This is because of the high internal resistance and the poor mechanical integrity of thick-film electrodes [8]. All cells with laser-structured thick-film electrodes are more stable than the ones without laser structuring. Thus, laser structuring of the thick-film electrodes is a viable approach for the high-energy battery [22].

#### 4.2.2. Cyclic Voltammetry

**Figure 8:**
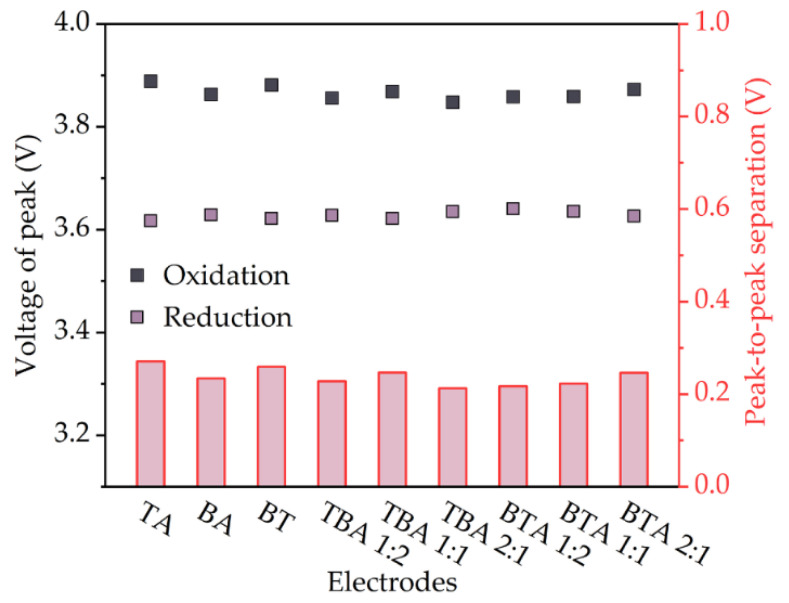
The voltages correspond to redox peaks and the separation of two peaks from CV measurement of cells with different electrodes.

The peaks measured in CV correspond to the oxidation/reduction of Ni^2+/^Ni^3+/^Ni^4+^ [20]. The position of the redox peaks depends on the component of the sample. Since TA and BA NMC 622 powders have the same chemical composition as determined by XRD, the redox peaks should locate at the same voltage. When there is a high barrier to electron transfer, electron transfer reactions are sluggish so that more positive (negative) potentials are required to observe oxidation (reduction) reactions, giving rise to larger differences in the potential between both peaks [33]. The cell with TBA 2:1 electrode has the minimum difference in the potential between both two peaks of 0.2 V, while the cell with TA electrode has the largest difference in the potential between both two peaks of 0.3 V, which indicates that thick-film TA electrode has a high barrier to electron transfer. The peaks reflect the insertion/deinsertion reactions that form solid solutions. These peaks indicate the Ni^2+/^Ni^3+/^Ni^4+^ oxidation/reduction reactions, which accompany the lithium insertion and deinsertion processes into/from the cathode materials [26]. Since Figure 8 shows that the redox peaks of all cells are located at the same position with only 0.2–0.3 V difference, this may indicate similar electrochemical kinetics of thick-film NMC 622 electrodes with different hierarchical structures.

#### 4.2.3. Electrochemical Impedance Spectroscopy

It is possible to obtain important values from each component in a LIBs from the EIS analysis [34]. R_SEI_ is the first semicircle in the Nyquist plot and associates with the SEI layer deposited on the electrode. R_ct_ is the second semicircle and relates to the kinetics of the electrochemical reaction, which is changed by the surface coating, phase transition, band gap structure, or particle size. The precise equivalent circuit model shown in Figure 6 is established to perform an EIS analysis. SEI resistance (R_SEI_) and charge transfer resistance (R_ct_) of cells with thick-film electrodes are obtained and shown in Table 3. Similar equivalent circuits are also used to describe the impedance response of the cathodes [29].

The value of R_SEI_ can be used to analyze the formation of the SEI layer, which is the result of the decomposition of the electrolyte, and the value of R_ct_ represents the faradic charge-transfer resistance and is used to clarify the reaction mechanism or temperature dependent characteristics of LIBs [34]. The cell with TA electrode shows the highest R_SEI_ of 11.5 Ω, which might be due to enlarged SEI film with increasing contact area of cathode with electrolyte and more side reactions [31]. The resistance of LIBs is affected by the cathodic charge transfer resistance [23]. On the contrary, bilayer cells with TBA 1:2, BTA 1:2, and BTA 2:1 have the lowest R_SEI_ at about 3 Ω. The electrode with TA displays the smallest R_ct_ value, while electrode with BT displays the highest R_ct_ value in comparison to electrodes with TA and BA. Bilayer electrode with BTA 2:1 displays the smallest R_ct_ value in comparison to the other bilayer electrodes and BT. Finally it can be concluded, that the bilayer electrode architecture can significantly influence the overall interfacial resistance.

## 5. Conclusions

Single-layer (TA, BA), blended (BT), and bilayer cathodes containing NMC 622 with two distinguishing particle sizes were manufactured. After drying and calendaring process, ultrafast laser ablation was applied to create channel structures in thick-film electrodes. Ultrafast laser ablation was used to generate groove structures inside electrode, in order to increase capacity retention at high C-rates. Rate capacity measurements, CV, and EIS were performed on all cells. Rate capability investigations show that the cell with TA electrodes owns the highest discharge capacity, while cells with bilayer electrodes exhibit different performances. Cells with BT display the highest capacity retention with 86% after 100 cycles. For cells with multilayer electrodes, the discharge capacity increases by 13% with increasing TA thickness (with small particle size) compared to cells with only BA at C/2. Cells with TA as bottom layer in bilayer electrodes (TBA) show higher capacity than cells containing bilayer electrode with TA on top. Cells with TBA 2:1 display the higher discharge capacity in bilayer electrodes at C/2. Besides, a layer with big particle size (BA) on the top can reduce mass loss resulted from laser structuring. Cells with laser-structured thick-film cathodes have higher discharge capacity than those with unstructured electrodes at C/2 to 5C. Only a slight peak shift in the order of 0.2–0.3 V was observed in redox peaks from CV measurements. The electrodes with hierarchical structures can decrease the interfacial resistance of coin cells. Cells with BA displays lower R_SEI_ than TA, while cells with TA own lower R_ct_. Cells with bilayer electrodes containing TBA 1:2 display the smallest R_SEI_. The electrochemical performance of cells containing hierarchical cathodes varies as a function of thickness of TA and BA.

## Figures and Tables

**Table 1:** The thickness, porosity, active mass loading, and areal capacity of electrodes consisted of different NMC 622 powder and with different multilayer structures.

Cathode	Thickness without Al Foil (µm)	Porosity (%)	Active Mass Loading (mg/cm^2^)	Areal Capacity (mAh/cm^2^)
TA thin	73 ± 1	35.1	18.2 ± 0.1	3.13 ± 0.02
BA thin	74 ± 1	35.3	18.4 ± 0.1	3.16 ± 0.02
BT thin	73 ± 1	35.2	18.1 ± 0.1	3.13 ± 0.02
TA thick	157 ± 1	35.3	38.9 ± 0.1	6.71 ± 0.01
BA thick	152 ± 1	35.4	37.6 ± 0.1	6.49 ± 0.02
BT thick	155 ± 2	35.3	38.5 ± 0.3	6.63 ± 0.05
TBA 1:2	152 ± 1	35.3	37.7 ± 0.2	6.50 ± 0.03
TBA 1:1	155 ± 1	35.2	38.5 ± 0.2	6.64 ± 0.03
TBA 2:1	153 ± 1	35.2	37.9 ± 0.1	6.53 ± 0.01
BTA 1:2	153 ± 1	35.2	38.0 ± 0.1	6.55 ± 0.02
BTA 1:1	151 ± 1	35.1	37.6 ± 0.1	6.47 ± 0.01
BTA 2:1	154 ± 1	35.1	38.3 ± 0.2	6.61 ± 0.03

**Table 2:** Active mass loading, areal capacity, and mass loss of laser-structured thick-film cathodes in comparison to unstructured ones.

Cathode (Laser Structured)	Active Mass Loading (mg/cm^2^)	Areal Capacity (mAh/cm^2^)	Mass Loss (%)
TA	34.3 ± 0.2	5.91 ± 0.04	11.2
BA	35.3 ± 0.1	6.09 ± 0.01	6.1
BT	35.8 ± 0.1	6.17 ± 0.02	6.6
TBA 1:2	35.6 ± 0.1	6.15 ± 0.02	5.4
TBA 1:1	36.1 ± 0.2	6.22 ± 0.03	6.2
TBA 2:1	35.8 ± 0.2	6.17 ± 0.03	5.5
BTA 1:2	34.1 ± 0.4	5.87 ± 0.02	10.3
BTA 1:1	34.5 ± 0.1	5.95 ± 0.01	8.1
BTA 2:1	36.3 ± 0.2	6.27 ± 0.01	5.1

**Table 3:** SEI resistance (R_SEI_) and charge transfer resistance (R_ct_) of cells containing thick-film electrodes.

R	TA	BA	BT	TBA 1:2	TBA 1:1	TBA 2:1	BTA 1:2	BTA 1:1	BTA 2:1
R_SEI_ (Ω)	11.5	4.5	6.5	3.0	9.2	8.6	3.3	7.3	3.1
R_ct_ (Ω)	6.7	9.1	42.4	15.3	36.7	23.8	39.2	16.2	8.5

## Supplementary Materials

The following are available online at https://www.mdpi.com/article/10.3390/nano11112962/s1, Figure S1: SEM images of the cross sections of thick-film electrodes with laser structures: (a) TA; (b) BA; (c) BT; (d) TBA 1:2; (e) TBA 1:1; (f) TBA 2:1; (g) BTA 1:2; (h) BTA 1:1 and (i) BTA 2:1. Figure S2: SEM images of BA-electrode close to a laser-ablated channel with (a) 2000 times and (b) 10,000 times magnification. Figure S3: SEM images of NMC 622 powders with (a) big particle size (BA) and (b) with small particle size (TA), with 20,000 magnification. The secondary particles and primary particles can be clearly distinguished.
