# Supplementary material for: Electrochemical Performance of Thick-Film Li(Ni0.6Mn0.2Co0.2)O2 Cathode with Hierarchic Structures and Laser Ablation
Source: Nanomaterials (Basel). 2021 Nov 4;11(11):2962. doi: 10.3390/nano11112962 (PMC8624508; doi:10.3390/nano11112962)
Supplement: Supplementary file 1 [file nanomaterials-11-02962-s001.zip › nanomaterials-1423488-supplementary.pdf]

Supplementary Materials

Electrochemical Performance of Thick-Film $Li(Ni_{0.6}Mn_{0.2}Co_{0.2})O_2$ Cathode with Hierarchic Structures and Laser Ablation

Zelai Song [1,2], Penghui Zhu [1,*], Wilhelm Pfleging [1] and Jiyu Sun [2]

1 Institute for Applied Materials—Applied Materials Physics (IAM-AWP), Karlsruhe Institute of Technology, Hermann-von-Helmholtz-Platz 1, 76344 Eggenstein-Leopoldshafen, Germany; songzelai@foxmail.com (Z.S.); wilhelm.pfleging@kit.edu (W.P.)

2 Key Laboratory of Bionic Engineering (Ministry of Education, China), Jilin University, Changchun 130022, China; sjy@jlu.edu.cn

* Correspondence: penghui.zhu@kit.edu

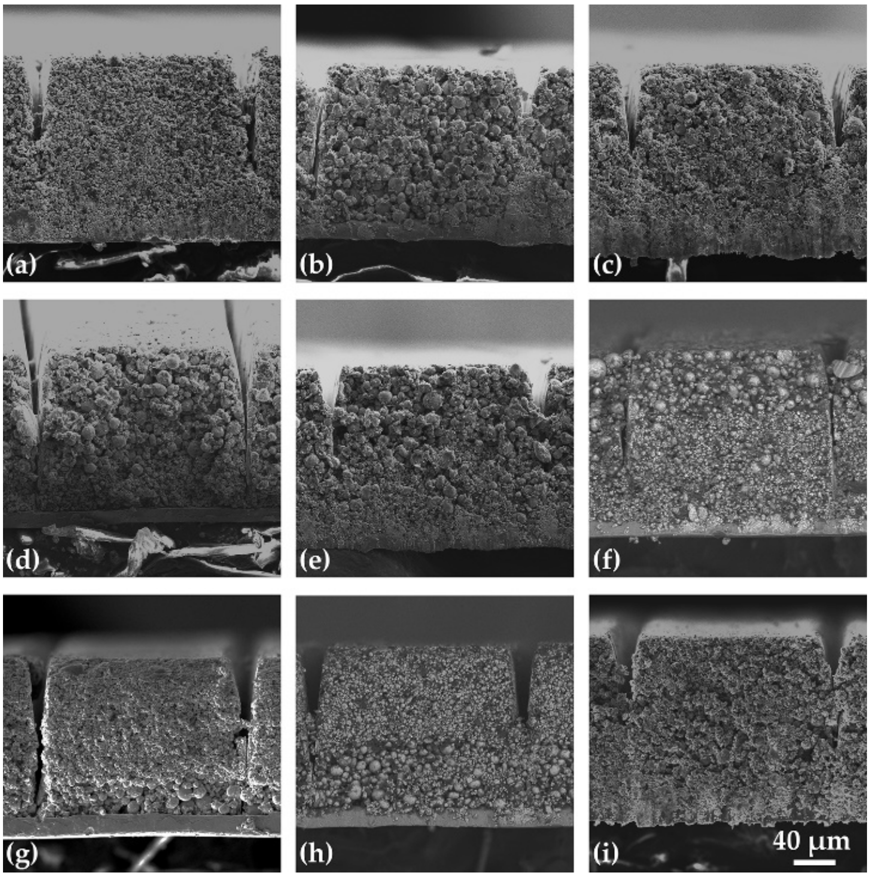

Figure S1. The cross sections of different thick-film electrodes with laser structures: (a) TA; (b) BA; (c) BT; (d) TBA 1:2; (e) TBA 1:1; (f) TBA 2:1; (g) BTA 1:2; (h) BTA 1:1 and (i) BTA 2:1.

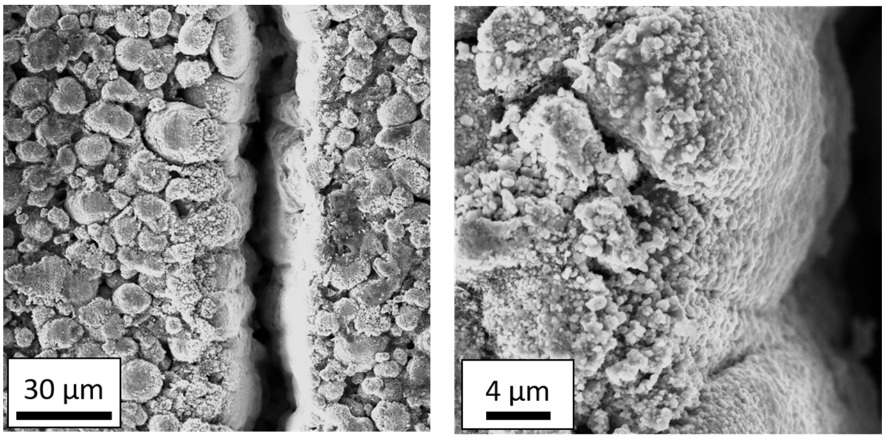

Figure S2. SEM images of BA-electrode close to a laser-ablated channel with (a) 2000 times and (b) 10000 times magnification.

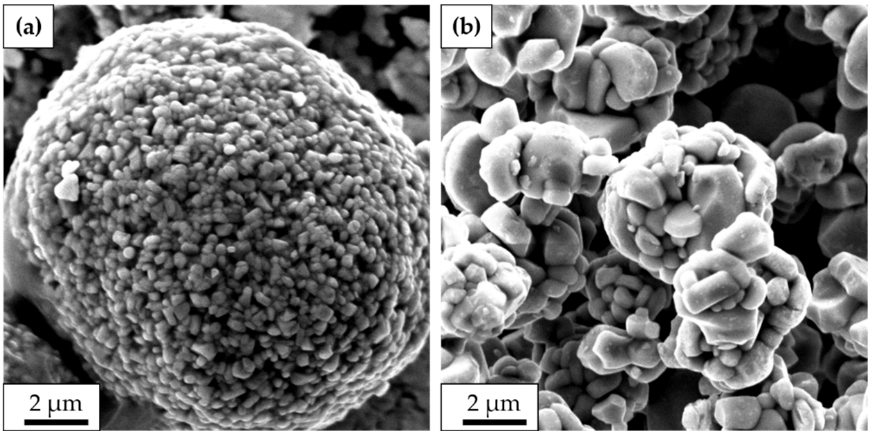

Figure S3. SEM images of NMC 622 powders with (a) big particle size (BA) and (b) with small particle size (TA), with 20000 magnification. The secondary particles and primary particles can be clearly distinguished.
